# *ACO2* clinicobiological dataset with extensive phenotype ontology annotation

**DOI:** 10.1038/s41597-021-00984-x

**Published:** 2021-08-05

**Authors:** Khadidja Guehlouz, Thomas Foulonneau, Patrizia Amati-Bonneau, Majida Charif, Estelle Colin, Céline Bris, Valérie Desquiret-Dumas, Dan Milea, Philippe Gohier, Vincent Procaccio, Dominique Bonneau, Johan T. den Dunnen, Guy Lenaers, Pascal Reynier, Marc Ferré

**Affiliations:** 1 Département d’Ophtalmologie, Centre Hospitalier Universitaire d’Angers, Angers, France; 2 Unité Mixte de Recherche MITOVASC, CNRS 6015/INSERM 1083, Université d’Angers, Angers, France; 3 Département de Biochimie et Génétique, Centre Hospitalier Universitaire d’Angers, Angers, France; 4 Genetics, and immuno-cell therapy Team, Mohammed First University, Oujda, Morocco; 5 Singapore National Eye Centre, Singapore Eye Research Institute, Duke-NUS, Singapore; 6 Human Genetics and Clinical Genetics, Leiden University Medical Centre, Leiden, The Netherlands

**Keywords:** Optic nerve diseases, Neurodegenerative diseases, Genetic databases

## Abstract

Pathogenic variants of the aconitase 2 gene (*ACO2*) are responsible for a broad clinical spectrum involving optic nerve degeneration, ranging from isolated optic neuropathy with recessive or dominant inheritance, to complex neurodegenerative syndromes with recessive transmission. We created the first public locus-specific database (LSDB) dedicated to *ACO2* within the “Global Variome shared LOVD” using exclusively the Human Phenotype Ontology (HPO), a standard vocabulary for describing phenotypic abnormalities. All the variants and clinical cases listed in the literature were incorporated into the database, from which we produced a dataset. We followed a rational and comprehensive approach based on the HPO thesaurus, demonstrating that *ACO2* patients should not be classified separately between isolated and syndromic cases. Our data highlight that certain syndromic patients do not have optic neuropathy and provide support for the classification of the recurrent pathogenic variants c.220C>G and c.336C>G as likely pathogenic. Overall, our data records demonstrate that the clinical spectrum of *ACO2* should be considered as a continuum of symptoms and refines the classification of some common variants.

## Abstract (summary)

**Table:** 

Measurement(s)	sequence_variant • Phenotypic variability
Technology Type(s)	DNA sequencing • Ophthalmologist
Factor Type(s)	sequence variant • phenotype
Sample Characteristic - Organism	Homo sapiens

Machine-accessible metadata file describing the reported data: 10.6084/m9.figshare.13574528

## Background & Summary

Aconitate hydratase (ACO2; EC# 4.2.1.3) is a ubiquitous human mitochondrial monomeric enzyme composed of 780 amino acids. It catalyses the second reaction of the citric acid cycle by isomerising citrate to isocitrate^1^. It is encoded by the aconitase 2 gene (*ACO2*; MIM# 100850) that extends over 35 kb on chromosome 22q13 and includes 18 translated exons^2^. Biallelic pathogenic variants of this gene have been associated with infantile cerebellar-retinal degeneration (ICRD; MIM# 614599), a severe neurodegenerative disorder with optic neuropathy beginning in childhood and including retinal dystrophy, cerebellar ataxia, seizure, strabismus, axial hypotonia and athetosis^3^. Isolated optic neuropathies have also been associated with *ACO2* pathogenic variants, either with recessive (locus *OPA9*; MIM# 616289)^4^ or dominant inheritance, as recently reported^5^. A recent review of the literature has listed a total of 26 individuals reported from 15 families with the syndromic form since 2012, while only seven individuals from four families have been reported with the recessive form of isolated optic neuropathy^6^. More specific clinical presentations, possibly without optic neuropathy, have also extended the clinical spectrum, such as a form of hereditary spastic paraplegia reported in two siblings^7^. The recent description by our team of 116 additional cases belonging to 94 families brings the total to over a hundred cases listed in the literature to date^5^.

The high frequency of dominant *ACO2* mutations in our molecular diagnosis experience of optic neuropathies and the diversity of the neurological involvement led us to develop a reliable database dedicated to *ACO2* as part of the Global Variome shared Leiden Open-source Variation Database (LOVD) installation^8,9^. Indeed, we recently developed such a database to list the genetic variants and clinical presentations of *OPA1*-related disorders, the major cause of dominant optic neuropathies with either isolated (80%; MIM# 165500) or syndromic (20%; MIM# 125250) presentations, and with some rare biallelic cases affected by early severe syndromes (MIM# 605390)^10^. The interoperability and the use of an international clinical thesaurus^11^ should, therefore, make it possible to progressively improve understanding of how this increasing number of genes responsible for optic neuropathies contributes to the diversity of the pathophysiological mechanisms responsible for their common clinical phenotype.

In this article, we describe the construction of this *ACO2* dataset, listing all the patients referenced in the literature, and drawing statistical information on gene variants and clinical diversity.

## Methods

### Nomenclature

All names, symbols, and OMIM numbers were checked for correspondence with current official names indicated by the Human Genome Organization (HUGO) Gene Nomenclature Committee^12^ and the Online Mendelian Inheritance in Man database (OMIM)^13^. The phenotype descriptions are based on the standardised Human Phenotype Ontology (HPO)^11^, indicating the HPO term name and identifier. *ACO2* variants are described according to both the NCBI genomic reference sequence NG_032143.1 and transcript reference sequence NM_001098.2, including 18 exons encoding a protein of 780 amino acids reference sequence NP_001089.1^14^. The numbering of the nucleotides reflects that of the cDNA, with “+1” corresponding to the “A” of the ATG translation initiation codon in the reference sequence, according to which the initiation codon is codon 1, as recommended by the version 2.0 nomenclature of the Human Genome Variation Society (HGVS; http://varnomen.hgvs.org)^15^. Information concerning changes in RNA levels has been added from the original papers or predicted from DNA mutations if not experimentally studied. Following the HGVS guidelines, deduced changes are indicated between brackets. Protein domains were predicted according to InterPro version 79.0^16^ and Pfam version 32.0^17^.

### Data collection

Since no locus-specific database (LSDB) dedicated to the *ACO2* gene previously existed (http://www.hgvs.org/locus-specific-mutation-databases, accessed on January 12, 2021), this work was done from scratch. The causative variants were collected from the literature published to date (January 2021)^3–7,18–24^ using the NCBI PubMed search tool^25^ with the keyword “*ACO2*,” and from the classifications of diagnostic laboratories in the Netherlands who recently decided to share them publicly (so-called the VKGL initiative)^26^. The positions of variants in the reference transcript were determined according to the HGVS nomenclature version 2.0^15^. Correct naming at the nucleotide and amino acid levels were verified, and reestablished when necessary, using the Mutalyzer 2.0.32 *Syntax Checker*^27^. All clinical descriptions of the dataset have been strictly and exhaustively translated using exclusively the HPO vocabulary, i.e. each phenotype mentioned having successfully matched an HPO term (or one of the synonyms associated with).

### Integrity of the dataset

The work was coordinated by a single ophthalmologist to ensure consistency, with the help of our clinical and research team specializing in hereditary optic neuropathies, which performs monitoring of literature on the subject for several years. No data was rejected, the corresponding molecular biologist or ophthalmologist were contacted when clarification was required. Finally, the consistency and integrity of the entire dataset was validated by the curator of the database, who is a referent specialist, before the technical validation that followed. (Please also refer to the section Author contributions.)

### Implementation of the dataset

The *ACO2* dataset belongs to the Global Variome shared Leiden Open-source Variation Database (LOVD) currently running under LOVD v.3.0 Build 23^9^, following the guidelines for LSDBs^28^ and hosted under the responsibility of the Global Variome/Human Variome Project^8^. The dataset reviews clinical and molecular data from patients carrying *ACO2* variants published in peer-reviewed literature, as well as unpublished contributions that are directly submitted.

### Data classification

**Fig. 1:**
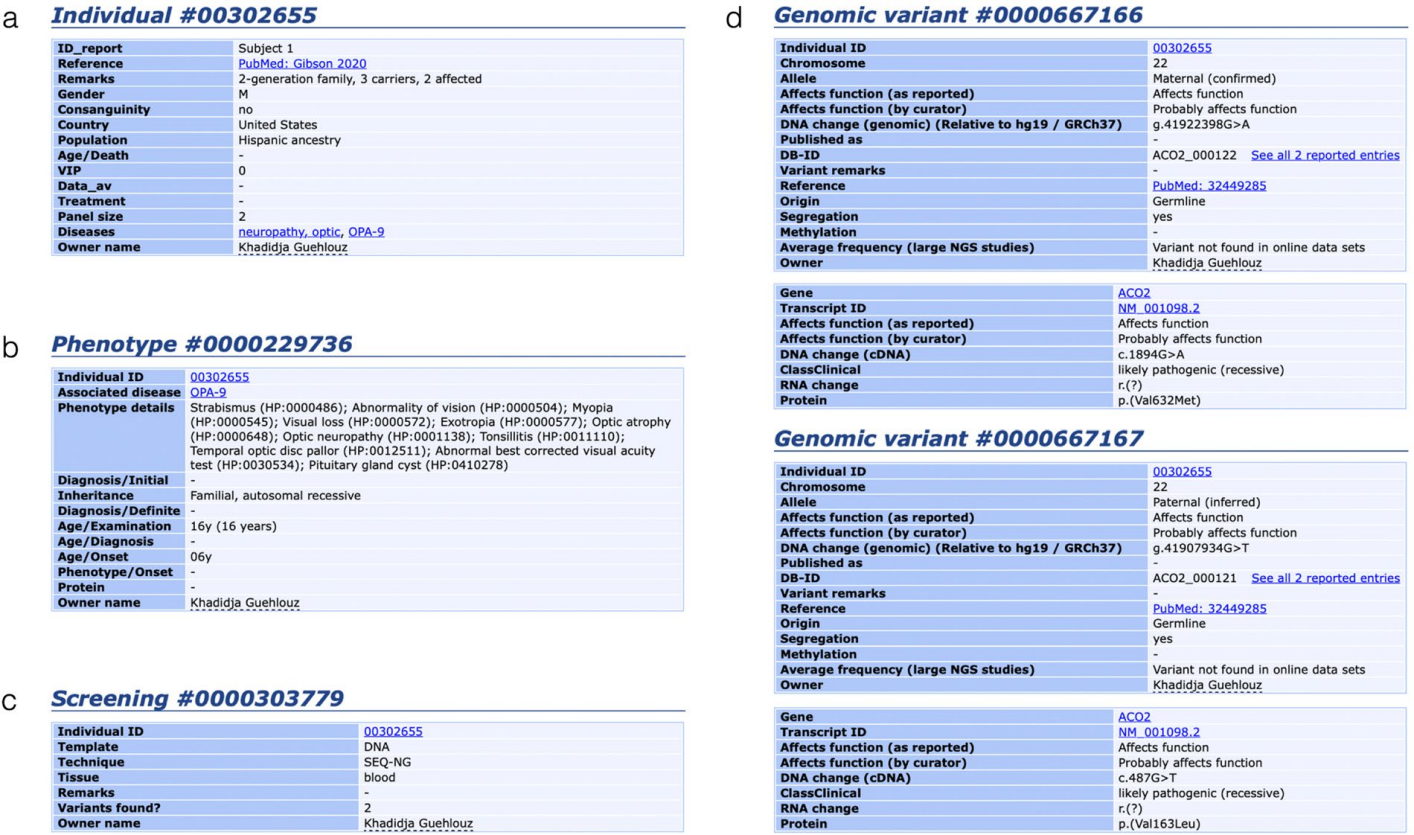
Sample recording for a given patient in the *ACO2* dataset. (**a**) individual items; (**b**) phenotype items; (**c**) screening items; and (**d**) molecular items (some uninformative lines were removed to save space). Abbreviations and legends of the fields are given by following the link *“Legend”* on the web page of each table; SEQ-NG: next generation sequencing; M: male. Data as of June 8, 2020.

The criteria of pathogenicity, which depend upon the clinical context and molecular findings, are stated under the headings: *“ClassClinical”* for the classification of the variant based on standardised criteria, and *“Affects function (as reported)”* and *“Affects function (by curator)”* respectively for the pathogenicity reported by the submitter and concluded by the curator (Fig. 1d). As several patients can be registered with different reported pathogenicity or new patients with existing variants added to the database, the status of the variants is reassessed on the basis of the data submitted and stated in the “*SUMMARY record*.”

### Dataset and analysis

Starting with version ACO2:200608 of the *ACO2* LSDB (last updated on June 8, 2020), we produced a dataset. To carry out the statistical analysis, the HPO terms have been checked and prepared using the suite of R packages ontologyX^29^ within R version 4.0.0^30^ to read in the OBO file version hp/releases/2020-06-08^11^. Hierarchical clustering is performed using the *hclust* function from the R-Core package^30^.

## Data Records

The *ACO2* dataset is available on the Code Ocean cloud-based computational reproducibility platform as a Code Ocean “compute capsule^31^”: snapshot of the *ACO2* dataset as of June 8, 2020, */data/LOVD_full_download_ACO2_2020-06-21_18.03.52.txt* (LOVD flat file format); the Human Phenotype Ontology data-version hp/releases/2020-06-08, */data/hp.obo.2020-06-08.txt* (OBO flat file format), which is also available in figshare^32^. The updated dataset is accessible on the Global Variome shared LOVD server (https://www.lovd.nl/ACO2; or through the Mitochondrial Dynamics variation portal: https://mitodyn.org). The data can also be retrieved via an application programming interface (API), i.e. a web service allowing simple queries and retrieval of basic gene and variant information (documentation available on the web page of the database)^9^; as well as serving as a public beacon in The Global Alliance for Genomics and Health Beacon Project^33^. General information is available on the database home page. The process for submitting data begins by clicking the *“Submit”* tab.

The *ACO2*-LOVD dataset contains four main interconnected tables. These tables are visible on a typical web page entry as shown in Fig. 1. The *“Individual”* table contains details of the patient examined, including gender, geographic origin, and patient identification, if applicable (Fig. 1a). The *“Phenotype”* table indicates the clinical phenotypic features, described according to the root of the Phenotypic abnormality subontology (HPO# HP:0000118; Fig. 1b). The *“Screening”* table gives details of the methods and techniques used for investigating the structural variants and the tissue analysed (Fig. 1c). The *“Variants”* table includes information about the sequence variations at the genomic (DNA) and the transcript variant (cDNA) levels, as well as the reported and concluded status for each variant (Fig. 1d). To date, the dataset records 123 patient records and 126 unique variants (Online-only Table 1).

Since the *ACO2* dataset is built on the same central platform (to allow interoperability) and on the same model (for ease of handling) as our previous *OPA1* gene database, the description of these data records extends our related work^10^, but the data recorded relates to a new gene and is entirely different.

## Technical Validation

### Molecular relevance

**Fig. 2:**
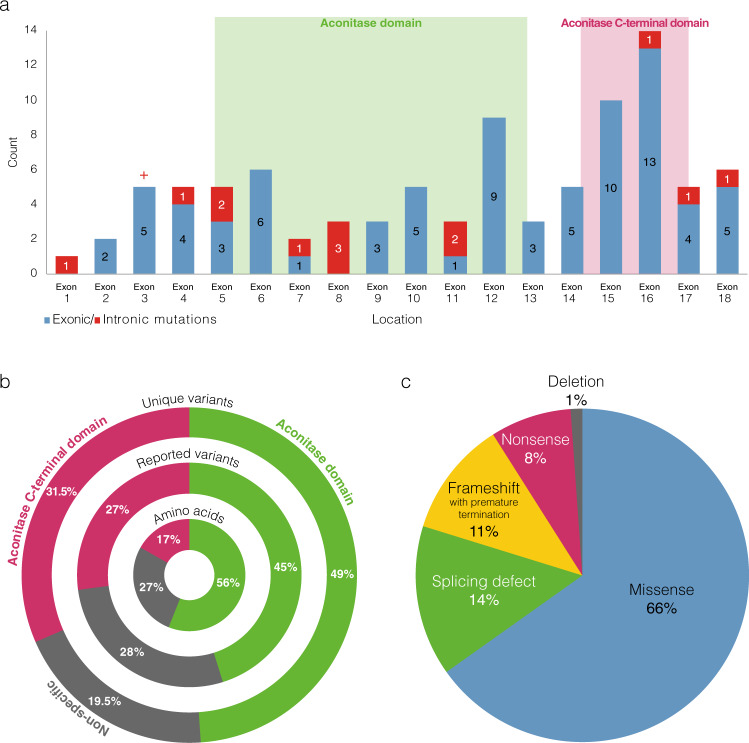
Distribution of the *ACO2* variants classified as pathogenic or likely pathogenic. (**a**) Distribution of the 89 unique variants. Exons involved in the variants are shown as blue bars; the variants in the intronic neighborhood of the exons are shown as red bars; the location of the two variants significantly more frequently reported being indicated by the “+” symbol. (**b**) Comparison for each region of its size on the sequence (Amino acids), of the distribution of the variants reports in the dataset (i.e. by counting each of the reported case of the same variant; Reported variants), and of the distribution of the unique variants (i.e. by counting only once several reported cases of the same variant). (**c**) Distribution of the different effects on the protein of the *ACO2* variants considered pathogenic. Data as of June 8, 2020.

The dataset contains 126 unique variants, of which 73% (92) are considered pathogenic sequence variants with almost two thirds in a dominant condition and the last third recessive, 7% (9) of unknown significance and 20% (25) benign (Online-only Table 1). The variants considered pathogenic, which affect the coding sequence and exon-intron boundaries of the gene (Fig. 2a), are particularly overrepresented in the aconitase C-terminal domain of the protein (spanning from end of exon 14 to half of exon 17), highlighting the importance of this domain in ACO2 functions (Fig. 2b), as well as the aconitase domain since it spans more than half of the protein sequence (from beginning of exon 5 to beginning of exon 13). Only one mutation considered pathogenic (intron 1 splicing site) and two benign (exon 2) are localized in the N-terminal presequence that is cleaved upon import of ACO2 into the mitochondrial matrix, predicted in the first 28 to 35 amino acids^34^. Among the most frequently observed pathogenic effects on ACO2, 66% are missense variants; 14% are associated with altered splicing, which produces effects that are difficult to assess experimentally; 11% are frameshift variants leading to a premature protein truncation; 8% are nonsense variations; one variant is a deletion of a single amino acid (Fig. 2c). Although only a few mutations are recurrent, two have been significantly more frequently reported, both located in exon 3 (Fig. 2a) with a recessive mode of inheritance: the c.220C>G variant, which induces a missense mutation p.(Leu74Val), has been reported 19 times affecting one allele (of which 14 belongs to compound heterozygous genotypes); the c.336C>G variant, which induces a missense mutation p.(Ser112Arg), has been reported 10 times affecting both alleles (homozygous).

The Global Variome shared LOVD server has integrated the data from The Genome Aggregation Database (gnomAD), which is the aggregation of the high-quality exome (protein-coding region) DNA sequence data for about 150,000 individuals^35^. It was decided to indicate for each variant its frequency in gnomAD rather than adding it as a new record, in order not to swamp the locus-specific databases (LSDBs) with data not associated to a phenotype. This information is mainly used to assist the curator in estimating the relevance of the classification of variants. In total, only two of the unique variants in our dataset (less than 2%) have assigned a *“likely pathogenic”* status with a frequency slightly below 1% in gnomAD. Interestingly, one of these, the previously mentioned c.220C>G variant, has a frequency from 0.2 to 0.4% (depending on versions of gnomAD) which does not allow its definitive classification as rare or frequent; it is registered in dbSNP (Build 153; dbSNP# rs141772938)^36^ referring to ClinVar (record last updated May 9, 2020; ClinVar# VCV000189310.8)^37^ for an unclear interpretation (*“Conflicting interpretations of pathogenicity”*). This last-mentioned heterozygous variant has been reported 19 times by at least five independent sources in our dataset, of which 14 are compound heterozygous disease cases. In addition, variant c.220C>G has so far not been reported as a third variant in a case with two other pathogenic variants. These observations provide strong evidence that has allowed us to classify this missense variant as “likely pathogenic (recessive)”, strengthening the importance of the LSDB approach and data sharing to support variant classification and DNA diagnostics.

### Clinical relevance

The dataset includes 123 patient records (71 males, 49 females, and three records of unspecified gender). Among these, 99 patients had isolated optic neuropathy^4–6,18^, 8 had infantile cerebellar-retinal degeneration (ICRD; MIM# 614559)^3^, 8 had various forms of encephalopathy (including epileptic encephalopathy and neonatal severe encephalopathy)^4,19,20^, 2 had spastic paraplegia^7^, together with single cases of autosomal recessive spinocerebellar ataxia, neurodegeneration^21^, retinal dystrophy (RDEOA; MIM# 617175)^22^, seizures^23^ and unclassified diseases^24^. Of these patients’ reports, 113 have an extended set of full clinical description (the remaining ten are either asymptomatic or not described), 90 relating to our Molecular Genetics Laboratory, along with data from 23 retrieved from publications. For the description of all of these phenotypes, use is made exclusively of a standard vocabulary for referencing phenotypic abnormalities, the so-called Human Phenotype Ontology (HPO)^11^, confirming the maturity of this ontology to describe eye diseases^38^. Genomic medicine requires the precise and standardized description of phenotypes^39–41^; these HPO annotations are key elements that make possible the development of algorithms for molecular diagnostics and genetic research.

**Fig. 3:**
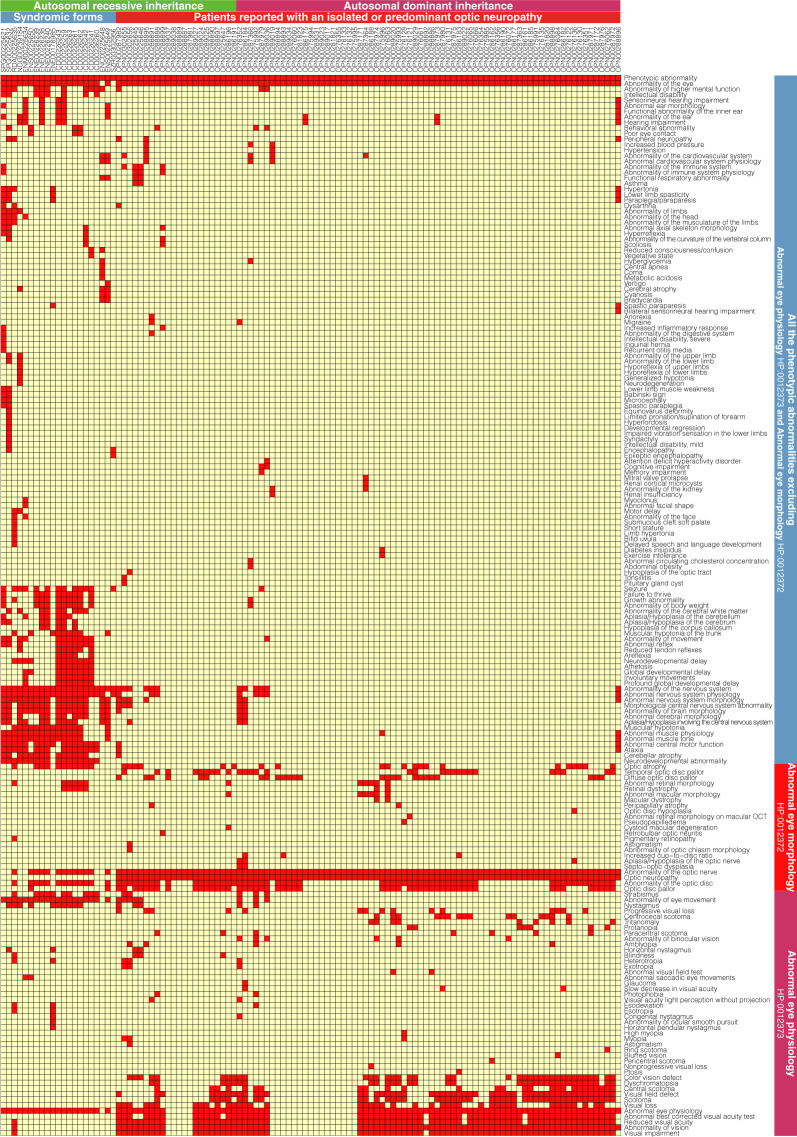
Visualisation of the Phenotypic abnormality subontology (HPO# HP:0000118) annotation in the *ACO2* dataset. Describing the 113 symptomatic patients’ reports with an extended set of full clinical description. Rows are clustered using *hclust* by separating the terms descending from Abnormal eye physiology (HPO# HP:0012373) and Abnormal eye morphology (HPO# HP:0012372); human readable shortened ontological term names were used (where possible). In columns, the identifiers of the patients (8 digits) are prefixed by code of the disease reported (3 letters): ICD: degeneration, cerebellar-retinal, infantile; RDA; dystrophy, retinal, with or without extraocular anomalies; ENM: encephalomyopathy, mitochondrial; ENC: encephalopathy; ENE: encephalopathy, epileptic; ENS: encephalopathy, neonatal, severe; NDG: neurodegeneration; OPN: neuropathy, optic; SPG: paraplegia, spastic; SZR: seizures. A red box indicates the presence of the phenotype. Data as of June 8, 2020.

**Fig. 4:**
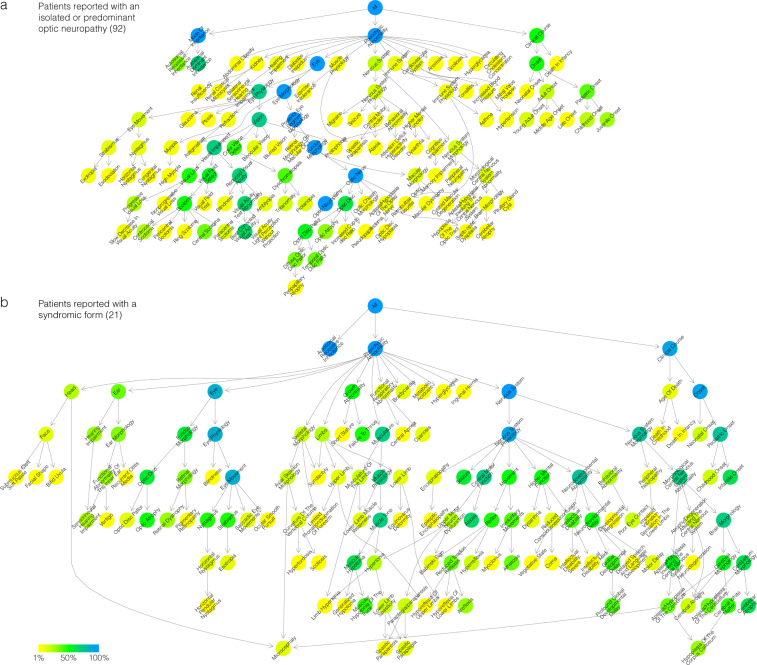
Visualisation of ontological annotation in the *ACO2* dataset by disease. Subgraphs of the mode of inheritance (HPO# HP:0000005), the phenotypic abnormalities (HPO# HP:0000118) and the natural history of the disease (Clinical Course, HPO# HP:0031797), descending from the root of all terms (All; HPO# HP:0000001) in the Human Phenotype Ontology: (**a**) for the 92 patients reported with an isolated or predominant optic neuropathy; (**b**) for the 21 remaining patients reported with a syndromic form. Terms which are annotated to exactly the same objects as well as all of their children have been removed, showing only informative terms. Arrows indicate relations between terms in the ontology. Colors correspond to the frequency of the phenotypes, from the least frequent in yellow to the most frequent in blue, the green color corresponding to a term present in half of the patients. Human readable shortened ontological term names were used (where possible). Data as of June 8, 2020.

A total of 154 unique HPO terms were used, each assigned from 1 to 92 patients for the most frequent term, optic neuropathy (HPO# HP:0001138); 208 unique HPO terms have been analysed by including the parent terms inferred by the ontological relationships. Figure 3 shows an exhaustive overview of the ontological annotation of the phenotypic abnormalities as a grid (mode of inheritance and natural history of the disease not shown), highlighting that patients reported with an isolated optic neuropathy, especially with a dominant mode of inheritance, have a similar limited phenotypic profile, different from the other patients whose phenotypic variability is particularly wide. We carried out the separate study of these two populations by showing the frequency of phenotypes and removing terms simply linking two terms together to focus the reading on informative phenotypes (Fig. 4): patients reported with an isolated or predominant optic neuropathy almost exclusively have a structural anomaly of the globe of the eye (Abnormal eye morphology; HPO# HP:0012372), in a predominantly dominant but also recessive mode of transmission, with an onset throughout life (Fig. 4a); the other patients reported with a syndromic form have rather a functional anomaly of the eye (Abnormal eye physiology; HPO# HP:0012373), in an exclusively recessive mode of transmission, with a beginning in the first years of life (Fig. 4b).

Overall, the extensive annotation using the phenotype ontology shows that the “isolated” versus “syndromic” separation is actually very relative: it is more likely to be a clinical spectrum with a continuum of symptoms. Figures 3 and 4 show that several cases reported as isolated are, in fact, affected by other symptoms; for the non-recurring symptoms, it is difficult to decide whether they are due to *ACO2* variants or whether they are just associated comorbidities. These figures also reveal that certain syndromic patients do not have optic neuropathy, which is therefore not compulsory in the *ACO2* phenotype as is the case for the *OPA1* gene, with only one exception published^42^.

## Usage Notes

The databases recording pathogenic variations, i.e. the so-called LSDBs, have proven to be the most complete because they rely on a curator who is a referent specialist for the gene or disease considered^43^. However, they are often based on isolated initiatives which use various interfaces, to the detriment of intuitiveness, and which are hosted on local servers, preventing their interoperability, unlike other types of databases which are central, i.e. encompassing all the genes of an organism, as in sequence databases^44,45^ or in databases oriented towards non-pathogenic variations^36,46^. With the aim to overcome this, the Human Variome Project currently favors the centralisation of LSDBs^8,47^.

Our current objective with the work reported here is to achieve a cluster of LOVD databases integrating the main genes responsible for optic neuropathies, whether isolated or syndromic, and with recessive or dominant transmission. Interoperability between these databases will be useful for molecular biologists analysing such panels of genes with, in particular, the possibility of detecting digenism. The *ACO2*-dataset, which is, to our knowledge, the only clinicobiological dataset dedicated to *ACO2*, interfaces molecular biology with medicine thanks to a common vocabulary, making it possible to link the phenotypic profiles of *ACO2* patients with those involving mutations in other genes or clinical presentations.

It will also allow a better understanding of the complex and overlapping relationships between isolated optic neuropathies and neurological syndromes involving optic neuropathy. Thus, this open-access dataset should prove useful for molecular biologists, researchers and clinicians.

## Data Availability

The source codes written in R programming language are available on the Code Ocean cloud-based computational reproducibility platform as a Code Ocean “compute capsule,” together with the dataset analysed in this article^31^: snapshot of the *ACO2* dataset as of June 8, 2020, */data/LOVD_full_download_ACO2_2020-06-21_18.03.52.txt* (LOVD flat file format); the Human Phenotype Ontology data-version hp/releases/2020-06-08, */data/hp.obo.2020-06-08.txt* (OBO flat file format). Thus, readers can reproduce and verify the results of this article without having to download or install anything. All the content is available under MIT License.

## Online-only Table

**Online-only Table 1:** Unique variants listed in the *ACO2* dataset (count: 126).

Region	DNA Variant^a^	RNA Variant^a^	Effect on Protein^a,b^	Classification of Variant^c^	Domain affected^d^	Rep.^e^	Database-ID^f^	References
Upstream	c.-504T>C	r.(?)	p.(=)	VUS	—	1	PHF5A_000001	^26^
Upstream	c.-494T>A	r.(?)	p.(=)	VUS	—	1	ACO2_000032	^26^
Intron 1	c.36+5del	r.spl?	p.?	Pathogenic (recessive)	—	1	ACO2_000084	^5^
Exon 2	c.72A>G	r.(?)	p.(=)	Benign	—	1	ACO2_000003	^26^
Exon 2	c.84A>G	r.(?)	p.(=)	Benign	—	1	ACO2_000033	^26^
Exon 2	c.166C>T	r.(?)	p.(Arg56Cys)	Pathogenic (dominant)	—	1	ACO2_000085	^5^
Exon 2	c.172C>T	r.(?)	p.(Arg58*)	Pathogenic (dominant)	—	1	ACO2_000050	^5^
Exon 3	c.180C>G	r.(?)	p.(Asn60Lys)	Likely benign	—	1	ACO2_000034	^26^
Exon 3	c.192A>C	r.(?)	p.(=)	Benign	—	2	ACO2_000004	^26^
Exon 3	c.196_197del	r.(?)	p.(Ser66Glyfs*10)	Pathogenic (dominant)	—	4	ACO2_000057	^5^
Exon 3	c.220C>G	r.(?)	p.(Leu74Val)	Likely pathogenic (recessive)	—	19	ACO2_000005	^4,18,24,26^
Exon 3	c.231C>T	r.(?)	p.(=)	Benign	—	1	ACO2_000006	^26^
Exon 3	c.275G>A	r.(?)	p.(Arg92Gln)	Pathogenic (dominant)	Aconitase	1	ACO2_000103	^5^
Exon 3	c.282C>T	r.(?)	p.(=)	Likely benign	Aconitase	2	ACO2_000007	^26^
Exon 3	c.316G>A	r.(?)	p.(Ala106Thr)	VUS	Aconitase	1	ACO2_000035	^26^
Exon 3	c.323T>C	r.(?)	p.(Leu108Pro)	Pathogenic (dominant)	Aconitase	1	ACO2_000051	^5^
Exon 3	c.336C>G	r.(?)	p.(Ser112Arg)	Pathogenic (recessive)	Aconitase	10	ACO2_000001	^3,19,20^
Intron 3	c.432+19T>C	r.(=)	p.(=)	Benign	Aconitase	1	ACO2_000008	^26^
Exon 4	c.441C>G	r.(?)	p.(Asn147Lys)	Likely pathogenic (dominant)	Aconitase	3	ACO2_000052	^5^
Exon 4	c.487G>A	r.(?)	p.(Val163Met)	Likely pathogenic (recessive)	Aconitase	1	ACO2_000104	^5^
Exon 4	c.487G>T	r.(?)	p.(Val163Leu)	Likely pathogenic (recessive)	Aconitase	2	ACO2_000121	^6^
Exon 4	c.514A>C	r.(?)	p.(Ile172Leu)	Pathogenic (dominant)	Aconitase	1	ACO2_000053	^5^
Intron 4	c.525+1G>A	r.spl?	p.?	Likely pathogenic (recessive)	Aconitase	1	ACO2_000105	^5^
Intron 4	c.525+6G>A	r.(=)	p.(=)	Likely benign	Aconitase	1	ACO2_000036	^26^
Intron 4	c.526-2A>G	r.spl?	p.?	Pathogenic (dominant)	Aconitase	1	ACO2_000054	^5^
Exon 5	c.573T>C	r.(?)	p.(=)	Benign	Aconitase	1	ACO2_000037	^26^
Exon 5	c.575A>G	r.(?)	p.(Asp192Gly)	Pathogenic (dominant)	Aconitase	1	ACO2_000055	^5^
Exon 5	c.610A>C	r.(?)	p.(Ile204Leu)	Likely pathogenic (recessive)	Aconitase	1	ACO2_000106	^5^
Exon 5	c.670C>T	r.(?)	p.(=)	Benign	Aconitase	1	ACO2_000038	^26^
Exon 5	c.680C>A	r.(?)	p.(Pro227His)	Pathogenic (dominant)	Aconitase	1	ACO2_000107	^5^
Intron 5	c.684+1G>T	r.spl?	p.?	Likely pathogenic (dominant)	Aconitase	1	ACO2_000120	^24^
Exon 6	c.718G>A	r.(?)	p.(Gly240Ser)	Pathogenic (dominant)	Aconitase	2	ACO2_000056	^5^
Exon 6	c.719G>C	r.(?)	p.(Gly240Ala)	Likely pathogenic (recessive)	Aconitase	5	ACO2_000002	^5,26^
Exon 6	c.740T>C	r.(?)	p.(Val247Ala)	Pathogenic (dominant)	Aconitase	1	ACO2_000058	^5^
Exon 6	c.741del	r.(?)	p.(Ile248Serfs*2)	Pathogenic (dominant)	Aconitase	3	ACO2_000059	^5^
Exon 6	c.776G>A	r.(?)	p.(Gly259Asp)	Likely pathogenic (recessive)	Aconitase	2	ACO2_000023	^4^
Exon 6	c.812G>A	r.(?)	p.(Gly271Asp)	Pathogenic (recessive)	Aconitase	1	ACO2_000108	^5^
Intron 6	c.836-2A>C	r.spl?	p.?	Likely pathogenic (dominant)	Aconitase	1	ACO2_000009	^26^
Exon 7	c.935G>A	r.(?)	p.(Arg312Gln)	Pathogenic (dominant)	Aconitase	1	ACO2_000060	^5^
Exon 8	c.978G>T	r.(?)	p.(Leu326Phe)	VUS	Aconitase	1	ACO2_000010	^26^
Exon 8	c.988C>T	r.(?)	p.(Pro330Ser)	Likely benign	Aconitase	1	ACO2_000011	^26^
Intron 8	c.1032+1G>C	r.spl?	p.?	Pathogenic (dominant)	Aconitase	1	ACO2_000061	^5^
Intron 8	c.1032+2T>C	r.spl?	p.?	Pathogenic (dominant)	Aconitase	1	ACO2_000062	^5^
Intron 8	c.1032+5G>A	r.spl?	p.?	Pathogenic (dominant)	Aconitase	2	ACO2_000063	^5^
Intron 8	c.1032+17C>T	r.(=)	p.(=)	Benign	Aconitase	2	ACO2_000012	^26^
Exon 9	c.1064C>G	r.(?)	p.(Pro355Arg)	Pathogenic (recessive)	Aconitase	2	ACO2_000086	^5^
Exon 9	c.1091T>C	r.(?)	p.(Val364Ala)	Likely pathogenic (recessive)	Aconitase	1	ACO2_000024	^22^
Exon 9	c.1113A>G	r.(?)	p.(=)	Likely benign	Aconitase	2	ACO2_000039	^26^
Exon 9	c.1132C>T	r.(?)	p.(Arg378*)	Pathogenic (dominant)	Aconitase	2	ACO2_000064	^5^
Exon 9	c.1181G>A	r.(?)	p.(Gly394Glu)	Pathogenic (recessive)	Aconitase	1	ACO2_000123	^23^
Exon 10	c.1237C>T	r.(?)	p.(Gln413*)	Pathogenic (dominant)	Aconitase	2	ACO2_000065	^5^
Exon 10	c.1240T>G	r.(?)	p.(Phe414Val)	Likely pathogenic (recessive)	Aconitase	2	ACO2_000025	^7^
Exon 10	c.1254dup	r.(?)	p.(Gly419Argfs*10)	Pathogenic (dominant)	Aconitase	1	ACO2_000066	^5^
Exon 10	c.1276A>G	r.(?)	p.(Thr426Ala)	VUS	Aconitase	1	ACO2_000013	^26^
Exon 10	c.1291G>A	r.(?)	p.(Gly431Ser)	Pathogenic (dominant)	Aconitase	1	ACO2_000067	^5^
Intron 10	c.1296+18C>T	r.(=)	p.(=)	Benign	Aconitase	1	ACO2_000014	^26^
Intron 10	c.1297-1G>C	r.spl?	p.?	Pathogenic (recessive)	Aconitase	1	ACO2_000109	^5^
Exon 11	c.1300C>T	r.(?)	p.(Gln434*)	Pathogenic (dominant)	Aconitase	1	ACO2_000068	^5^
Intron 11	c.1370+1G>A	r.spl?	p.?	Pathogenic (dominant)	Aconitase	1	ACO2_000069	^5^
Exon 12	c.1387G>C	r.(?)	p.(Gly463Arg)	Pathogenic (dominant)	Aconitase	1	ACO2_000070	^5^
Exon 12	c.1390G>T	r.(?)	p.(Glu464*)	Pathogenic (recessive)	Aconitase	1	ACO2_000087	^5^
Exon 12	c.1406T>A	r.(?)	p.(Val469Asp)	Pathogenic (recessive)	Aconitase	1	ACO2_000110	^5^
Exon 12	c.1411T>C	r.(?)	p.(Ser471Pro)	Pathogenic (dominant)	Aconitase	1	ACO2_000071	^5^
Exon 12	c.1420A>G	r.(?)	p.(Arg474Gly)	Pathogenic (dominant)	Aconitase	1	ACO2_000072	^5^
Exon 12	c.1425C>G	r.(?)	p.(Asn475Lys)	Pathogenic (dominant)	Aconitase	1	ACO2_000073	^5^
Exon 12	c.1444G>A	r.(?)	p.(Ala482Thr)	Pathogenic (dominant)	Aconitase	1	ACO2_000111	^5^
Exon 12	c.1452_1459del	r.(?)	p.(Glu485Cysfs*44)	Pathogenic (dominant)	Aconitase	1	ACO2_000074	^5^
Exon 12	c.1454A>G	r.(?)	p.(Glu485Gly)	Pathogenic (dominant)	Aconitase	1	ACO2_000075	^5^
Exon 13	c.1508G>A	r.(?)	p.(Gly503Glu)	Pathogenic (recessive)	Aconitase	1	ACO2_000112	^5^
Exon 13	c.1534G>A	r.(?)	p.(Asp512Asn)	Likely pathogenic (recessive)	—	1	ACO2_000046	^21^
Exon 13	c.1561A>T	r.(?)	p.(Lys521*)	Pathogenic (dominant)	—	1	ACO2_000113	^5^
Exon 13	c.1581G>A	r.(?)	p.(=)	Benign	—	1	ACO2_000015	^26^
Intron 13	c.1605+3G>A	r.spl?	p.?	Likely benign	—	2	ACO2_000016	^26^
Exon 14	c.1671C>T	r.(?)	p.(=)	Likely benign	—	1	ACO2_000040	^26^
Exon 14	c.1690C>T	r.(?)	p.(Arg564Cys)	Likely pathogenic (recessive)	—	1	ACO2_000114	^5^
Exon 14	c.1722G>C	r.(?)	p.(Trp574Cys)	Pathogenic (recessive)	—	1	ACO2_000124	^23^
Exon 14	c.1724dup	r.(?)	p.(Asp575Glufs*21)	Pathogenic (dominant)	—	1	ACO2_000076	^5^
Exon 14	c.1753_1755del	r.(?)	p.(Leu585del)	Pathogenic (dominant)	Aconitase C-term	1	ACO2_000088	^5^
Exon 14	c.1753_1759del	r.(?)	p.(Leu585Argfs*73)	Pathogenic (dominant)	Aconitase C-term	1	ACO2_000089	^5^
Exon 15	c.1787A>C	r.(?)	p.(His596Pro)	Pathogenic (dominant)	Aconitase C-term	1	ACO2_000077	^5^
Exon 15	c.1789A>T	r.(?)	p.(Ile597Phe)	Pathogenic (dominant)	Aconitase C-term	1	ACO2_000078	^5^
Exon 15	c.1808G>C	r.(?)	p.(Trp603Ser)	Pathogenic (dominant)	Aconitase C-term	2	ACO2_000090	^5^
Exon 15	c.1819C>T	r.(?)	p.(Arg607Cys)	Likely pathogenic (recessive)	Aconitase C-term	1	ACO2_000026	^19^
Exon 15	c.1820G>A	r.(?)	p.(Arg607His)	Pathogenic (dominant)	Aconitase C-term	1	ACO2_000079	^5^
Exon 15	c.1859G>A	r.(?)	p.(Gly620Asp)	Likely pathogenic (recessive)	Aconitase C-term	2	ACO2_000021	^20^
Exon 15	c.1877dup	r.(?)	p.(Asn626Lysfs*20)	Pathogenic (dominant)	Aconitase C-term	1	ACO2_000080	^5^
Exon 15	c.1879G>A	r.(?)	p.(Gly627Ser)	Pathogenic (recessive)	Aconitase C-term	1	ACO2_000091	^5^
Exon 15	c.1894G>A	r.(?)	p.(Val632Met)	Likely pathogenic (recessive)	Aconitase C-term	2	ACO2_000122	^6^
Exon 15	c.1898G>A	r.(?)	p.(Arg633His)	Pathogenic (dominant)	Aconitase C-term	2	ACO2_000092	^5^
Intron 15	c.1954-12C>T	r.(=)	p.(=)	Benign	Aconitase C-term	1	ACO2_000017	^26^
Exon 16	c.1981G>A	r.(?)	p.(Gly661Arg)	Pathogenic (recessive)	Aconitase C-term	1	ACO2_000093	^5^
Exon 16	c.1981G>C	r.(?)	p.(Gly661Arg)	Likely pathogenic (recessive)	Aconitase C-term	1	ACO2_000027	^4^
Exon 16	c.1986C>T	r.(?)	p.(=)	Likely benign	Aconitase C-term	2	ACO2_000048	^26^
Exon 16	c.1987G>T	r.(?)	p.(Glu663*)	Pathogenic (dominant)	Aconitase C-term	1	ACO2_000081	^5^
Exon 16	c.1995dup	r.(?)	p.(Gly666Argfs*46)	Pathogenic (dominant)	Aconitase C-term	1	ACO2_000094	^5^
Exon 16	c.1997G>C	r.(?)	p.(Gly666Ala)	Likely pathogenic (recessive)	Aconitase C-term	1	ACO2_000047	^21^
Exon 16	c.1999G>A	r.(?)	p.(Glu667Lys)	Pathogenic (dominant)	Aconitase C-term	1	ACO2_000082	^5^
Exon 16	c.2006C>T	r.(?)	p.(Ser669Leu)	Pathogenic (dominant)	Aconitase C-term	5	ACO2_000083	^5^
Exon 16	c.2011C>T	r.(?)	p.(Arg671Trp)	Pathogenic (dominant)	Aconitase C-term	1	ACO2_000095	^5^
Exon 16	c.2012G>A	r.(?)	p.(Arg671Gln)	Pathogenic (dominant)	Aconitase C-term	5	ACO2_000041	^5,26^
Exon 16	c.2035C>T	r.(?)	p.(Arg679Cys)	Pathogenic (dominant)	Aconitase C-term	1	ACO2_000096	^5^
Exon 16	c.2048G>A	r.(?)	p.(Gly683Asp)	Pathogenic (recessive)	Aconitase C-term	1	ACO2_000115	^5^
Exon 16	c.2048G>T	r.(?)	p.(Gly683Val)	Likely pathogenic (recessive)	Aconitase C-term	2	ACO2_000022	^20^
Exon 16	c.2050C>T	r.(?)	p.(Arg684Trp)	VUS	Aconitase C-term	1	ACO2_000042	^26^
Exon 16	c.2057T>G	r.(?)	p.(Ile686Ser)	Pathogenic (dominant)	Aconitase C-term	1	ACO2_000116	^5^
Exon 16	c.2069G>A	r.(?)	p.(Ser690Asn)	Likely benign	Aconitase C-term	1	ACO2_000049	^26^
Intron 16	c.2086+2T>C	r.spl?	p.?	Pathogenic (dominant)	Aconitase C-term	1	ACO2_000097	^5^
Exon 17	c.2107G>C	r.(?)	p.(Gly703Arg)	Pathogenic (dominant)	Aconitase C-term	1	ACO2_000098	^5^
Exon 17	c.2128G>C	r.(?)	p.(Ala710Pro)	Pathogenic (dominant)	Aconitase C-term	1	ACO2_000099	^5^
Exon 17	c.2135C>T	r.(?)	p.(Pro712Leu)	Likely pathogenic (recessive)	Aconitase C-term	1	ACO2_000028	^19^
Exon 17	c.2208G>C	r.(?)	p.(Lys736Asn)	Likely pathogenic (recessive)	—	1	ACO2_000029	^4^
Intron 17	c.2208+1dup	r.spl?	p.?	VUS	—	2	ACO2_000030	^18^
Intron 17	c.2208+1G>A	r.spl?	p.?	Pathogenic (dominant)	—	1	ACO2_000117	^5^
Intron 17	c.2208+11C>T	r.(=)	p.(=)	Benign	—	1	ACO2_000043	^26^
Intron 17	c.2209-1G>A	r.spl?	p.?	Pathogenic (recessive)	—	1	ACO2_000100	^5^
Exon 18	c.2212_2213del	r.(?)	p.(Leu738Glufs*26)	Likely pathogenic (recessive)	—	4	ACO2_000118	^5^
Exon 18	c.2237A>G	r.(?)	p.(Asn746Ser)	VUS	—	1	ACO2_000044	^26^
Exon 18	c.2265C>T	r.(?)	p.(=)	Benign	—	1	ACO2_000018	^26^
Exon 18	c.2278G>A	r.(?)	p.(Glu760Lys)	Likely benign	—	1	ACO2_000019	^26^
Exon 18	c.2282C>T	r.(?)	p.(Thr761Met)	Pathogenic (recessive)	—	1	ACO2_000119	^5^
Exon 18	c.2293T>G	r.(?)	p.(Trp765Gly)	Pathogenic (dominant)	—	1	ACO2_000101	^5^
Exon 18	c.2311G>C	r.(?)	p.(Ala771Pro)	Pathogenic (dominant)	—	2	ACO2_000102	^5^
Exon 18	c.2328_2331del	r.(?)	p.(Lys776Asnfs*49)	Likely pathogenic (recessive)	—	1	ACO2_000031	^22^
Exon 18	c.2334G>A	r.(?)	p.(=)	Benign	—	1	ACO2_000045	^26^
3′-UTR^g^	c.*3G>A	r.(=)	p.(=)	Benign	—	1	ACO2_000020	^26^
Downstream	c.*2157T>C	r.(=)	p.(=)	VUS	—	1	POLR3H_000001	^26^

*Note*: Data as of June 8, 2020; for more information, please refer to the database using Database-ID.

^a^Mutational data are described using the nomenclature of the Human Genome Variation Society (http://www.hgvs.org/mutnomen). Nucleotide numbering reflects cDNA numbering with “+1” corresponding to the A of the ATG translation initiation codon in the *ACO2* reference sequence (RefSeq: NM_001098.2), according to journal guidelines. The initiation codon is codon 1.

^b^Predicted effect on protein based on clinical consequences.

^c^Classification of variant based on clinical consequences, using standardized criteria: pathogenic (disease-associated), likely pathogenic (likely disease-associated), VUS (variant of unknown significance), likely benign (likely not disease-associated), benign (not disease-associated); including inheritance (dominant or recessive) if applicable.

^d^Affected domain of the protein: aconitase domain (from start of exon 3 to start of exon 13); aconitase C-terminal (aconitase C-term, from end of exon 14 to half of exon 17).

^e^Number of times variant has been reported in the database.

^f^Identifier of variant in the *ACO2* database (https://www.lovd.nl/ACO2).

^g^Three prime untranslated region.
